# Genome Sequence of Lactobacillus fermentum 47-7, a Good *In Vitro* Probiotic Strain Isolated from a Healthy Thai Infant

**DOI:** 10.1128/MRA.01014-19

**Published:** 2019-09-26

**Authors:** Atthaphon Konyanee, Panjamaporn Yotpanya, Marutpong Panya, Chulapan Engchanil, Namfon Suebwongsa, Wises Namwat, Hlainghlaing Thaw, Kiatichai Faksri, Nipaporn Sankuntaw, Viraphong Lulitanond

**Affiliations:** a Research and Diagnostic Center for Emerging Infectious Diseases, and Department of Microbiology, Faculty of Medicine, Khon Kaen University, Khon Kaen, Thailand; b College of Medicine and Public Health, Ubon Ratchathani University, Ubon Ratchathani, Thailand; c Chulabhorn International College of Medicine, Thammasat University, Pathum Thani, Thailand; d Department of Microbiology, University of Medicine, Magway, Magway, Myanmar; e School of Medicine, Walailak University, Nakhon Si Thammarat, Thailand; University of Rochester School of Medicine and Dentistry

## Abstract

We report the genome sequence of Lactobacillus fermentum 47-7, a good *in vitro* probiotic strain isolated from an infant. Its genome size is 1.83 Mb, it is assembled from 180 contigs, and it consists of 1,636 protein-coding genes, 15 rRNAs, 57 tRNAs, and 4 noncoding RNAs. This genome sequence will be useful for a variety of applications.

## ANNOUNCEMENT

Lactobacillus fermentum belongs to the genus Lactobacillus, phylum *Firmicutes*. Several strains of *L. fermentum* have been used as probiotics (1–3). Our 47-7 strain was isolated from a fecal specimen from a healthy infant on de Man-Rogosa-Sharpe (MRS) agar and was finally identified as Lactobacillus fermentum by 16S rRNA gene sequencing. This strain was demonstrated to exhibit good *in vitro* probiotic properties (4) and was subjected to genome sequencing for further studies and applications.

*L. fermentum* 47-7 cultured for 24 h at 37°C in MRS broth was subjected to genomic DNA (gDNA) extraction using the Gentra Puregene Yeast/Bact kit (Qiagen). The sequencing library was prepared using a TruSeq DNA PCR-free library preparation kit and sequenced with a HiSeq 2000 platform (Illumina, USA), with paired-end read lengths of 101 bp; the other sequencing library was prepared with the Ion Xpress Plus gDNA fragment library preparation kit and Ion PGM Template OT2 200 kit and sequenced with the Ion Torrent Personal Genome Machine (PGM) platform (Thermo Fisher Scientific, USA) using an Ion 314 Chip with a single-end read length of 200 bp. The FASTQ sequence files were generated, and the total raw reads are 10,293,934 (HiSeq 2000) and 583,615 (PGM). Default parameters were used for all software unless otherwise specified. The FASTQ sequence files were processed for quality trimming and were filtered using the FASTX-Toolkit (v 0.0.13), with setting parameters of -q minBaseQ >20 (5), and the quality assessment was performed with FastQC (v 0.11.4) (6). The number of filtered reads of HiSeq 2000 and PGM are 9,340,868 and 488,674, respectively, and were aligned separately with the reference genome of Lactobacillus fermentum IFO 3956 (NCBI RefSeq accession number NC_010610) using the Burrows-Wheeler Aligner (BWA) MEM algorithm (v 0.7.5) (http://bio-bwa.sourceforge.net/) to create Sequence Alignment Map (SAM) files. The SAM files (HiSeq 2000 and PGM) were converted and merged to a binary alignment map (BAM) file by using Picard tools (v 2.5.0) (http://broadinstitute.github.io/picard/). The merged BAM file was subjected to local realignment and base quality score recalibration (BQSR), performed using the Genome Analysis Toolkit (GATK) (v 3.3) (7–9). The merged BAM file was used for reference-based genome assembly by using SAMtools (v 0.1.19) (10), Bcftools (11), and vcfutils (12), with setting parameters -Q minBaseQ >30, -q minMapQ >30, -C adjustMQ >50, -E redoBAQ and -d minDepth >20. The genome sequence of *L. fermentum* 47-7 consists of 1,830,930 bp (7,140,640 high-quality reads [Q20 or higher] aligned with an 87.23% coverage region of *L. fermentum* IFO 3956) in 180 contigs, with a high genome coverage of 411.1×, an *N*_50_ value of 37,781 bp, and GC content of 52.2%. Genome annotation and gene prediction were performed using the NCBI Prokaryotic Genome Annotation Pipeline (PGAP) server (13). The annotation revealed 1,636 protein-coding genes and a total of 72 RNAs, including 57 tRNAs and 5 rRNA gene operons (5S, 16S, and 23S rRNAs). Genes for bile hydrolysis and amino acid permease probably associated with the survival ability of 47-7 in the gastrointestinal environment (14, 15), a gene cluster for riboflavin production, i.e., *ribG*, *ribB*, *ribA,* and *ribH* (16), and a gene coding for a colicin V biosynthesis protein were found.

### Data availability.

This whole-genome shotgun project has been deposited in DDBJ/EMBL/GenBank under the accession number CP017712. The SRA/DRA/ERA accession numbers are SRX2239653 and SRX2239654. The BioSample and BioProject numbers are SAMN05893390 and PRJNA347617, respectively.
